# Still No Substantial Evidence to Use Prophylactic Antibiotic at Operative Vaginal Delivery: Systematic Review and Meta-Analysis

**DOI:** 10.1155/2020/1582653

**Published:** 2020-05-19

**Authors:** Yifru Berhan, Sisay Kirba, Achamyelesh Gebre

**Affiliations:** St. Paul's Hospital Millennium Medical College, Addis Ababa, Ethiopia

## Abstract

**Background:**

Postpartum maternal infection is still a common problem worldwide, mainly due to obstetric risk factors. The use of prophylactic antibiotic at operative vaginal delivery (OVD), taking it as a standalone risk factor, has been controversial. The purpose of this review was to rigorously evaluate the association of OVD with postpartum infection and shed light on such highly controversial issue.

**Methods:**

A computer-based literature search was done mainly in the databases of PUBMED, HINARI health research, and the Cochrane library. Systematic review and meta-analysis were done by including 14 articles published between 1990 and August 2019.

**Results:**

The average absolute risk of postpartum infection at OVD from seven large cohort studies was 1%. Few studies showed a weak association of OVD with postpartum infection without being adjusted to perineal wound, but the pooled meta-analysis showed statistically significant association with non-OVD. In the included randomized trial, 97% of the study participants had perineal wound for whom repairs were performed; the risks of maternal infection and perineal wound breakdown were comparable, and maternal infections other than perineal wound infection did not show significant difference between prophylactic antibiotic and placebo groups. The majority of included studies demonstrated a strong association of postpartum infection and perineal wound dehiscence with episiotomy and perineal tear.

**Conclusion:**

Both the relative and absolute risks of postpartum infection at OVD are extremely low unless accompanied by episiotomy and 3^rd^/4^th^t degree perineal tear. From previous studies, there is no substantial evidence to use prophylactic antibiotic at OVD, but episiotomy and perineal tear.

## 1. Introduction

Postpartum maternal infection is still a common problem worldwide, but the exact incidence is unknown because of the occurrence of the majority of infections after discharge from a health facility [1, 2]. Depending on the underlying risk factor, the quality of obstetric care, and the region where the women live in, postpartum infection may complicate 1% to 4% of vaginal and 5% to 20% of caesarean deliveries [1, 3–5]. A large body of evidence demonstrated that the single most important risk factor for postpartum infection is caesarean section (CS) [6].

There are several other risk factors associated with postpartum infection (like bacterial vaginosis, prolonged rupture of membranes, chorioamnionitis, and manual removal of the placenta), for which antibiotic administration is important, but the use of prophylactic antibiotic at operative vaginal delivery (OVD), taking it as a standalone risk factor, has been very controversial for decades. The controversy is with regard to the difference in potential risk abdominal and vaginal operative deliveries incurring to postpartum infection.

Traumatic or surgical wound in the genital tract and perineum increases the risk of postpartum infection, which is again the common cause for wound infection and dehiscence [7]. The higher risk of postpartum infection (endometritis in particular) in women with CS is also mainly due to the surgical entry into the endometrial cavity. Similarly, since performing episiotomy and sustaining trauma to the genital tract and perineum during OVD are common, there is a tendency to associate the increased risk of postpartum infection with OVD. Taking the less presumed risk, many used to disagree with the routine administration of antibiotic prophylaxis at OVD. Among others, the World Health Organization (WHO) and many national guidelines are not recommending prophylactic antibiotic at OVD [8–11].

The argument is that repaired episiotomy or perineal tear wound infection and dehiscence is due to the surgical and traumatic wound, not merely by the applied instruments. In contrast, all CSs cause surgical wound to the uterus and abdominal wall, but all OVDs are not conducted with episiotomy and do not always cause trauma to the genital tract and perineum. The significant reduction of perineal surgical wound infection with prophylactic antibiotic is also an indirect evidence for the increased risk of infection in women with perineal wound [6, 12].

In general, there is a paucity of data on the importance of prophylactic antibiotics at OVD. The first randomized clinical trial was done in 1989 with small sample size (393 study participants) for which the updated Cochrane review in 2017 concluded that the data were inadequate to make conclusion [13].

The second randomized clinical trial was reported in May 2019 and made a firm conclusion on the importance of prophylactic antibiotic at OVD [14] and got much of the media attention, but the strength of the evidence for that purpose is questionable. The few observational studies that reported the risk of postpartum infection in relation to OVD were not reviewed from the perspective of the contribution of perineal wound to postpartum infection. Therefore, the purpose of this review was to rigorously evaluate the association of OVD with postpartum infection as a standalone risk factor and shed light on such highly controversial issue.

## 2. Methods

### 2.1. Search Strategy

A computer-based literature search was done in the databases of Medline/PUBMED, HINARI health research, and the Cochrane library. Further search was conducted using Google scholar search engine and from the reference lists of retrieved articles. The search terms were as follows: operative vaginal delivery, instrumental vaginal delivery, forceps assisted delivery, vacuum assisted delivery, obstetric forceps, vacuum extractor, ventouse assisted delivery, antibiotic prophylaxis, postpartum infection (as listed below), maternal sepsis, puerperal sepsis, perineal wound infection, perineal tear, perineal wound, perineal wound dehiscence, and episiotomy. Boolean logic (and/or) was applied during searching by combining the search terms alternatively.

### 2.2. Study Selection and Inclusion Criteria

In this review, the literature search was done independently by two authors (YB and SK) using the selected search terms. When there was a discrepancy in individually selected studies, it was resolved by discussion and by reviewing those specific studies in detail, together with the third author. The predetermined inclusion criteria were studies that (1) compared the risk of postpartum infection with or without antibiotic prophylaxis in instrument assisted vaginal deliveries, (2) reported the magnitude of postpartum infection among women for whom vacuum or forceps or both applied, (3) were written in English, and (4) were published between 1990 and August 2019. Some of the studies were included only for their descriptive data.

### 2.3. Data Extraction

Many of the included studies reported several other relevant data. Since the interest of this review was on the potential risk of OVD to postpartum infection and the place of prophylactic antibiotic for it, the abstracted data were limited to those variables. Standard Excel spreadsheet was used to extract the required data from selected individual studies. The following data were extracted from the selected studies: name of authors, study period, study design, study location, study population/sample size, risk factors, statistical analysis, postpartum infections cross tabulated with prophylactic antibiotic, OVD, forceps or vacuum delivery, and episiotomy and/or perineal tear.

### 2.4. Study Quality Assessment

The risk of bias in the randomized clinical trial [14] was assessed using the Cochrane tool. STROBS (Strengthening the Reporting of Observational Studies) checklist, as recommended by the WHO [15], was used to assess the risk of bias in the included observational studies. Starting from the title and abstract to the discussion section, an assessment was made with 21 defined items. In their introduction section, the rationale and study objective, in their methods section (as summarized in Table 1), the study population/sample size, study design, study period, outcome measures and statistical methods were described. In the results section, the descriptive data, outcome, and the main results were assessed. In the discussion section, the descriptive data of all and conclusions drawn from them are appropriate. However, we have identified that the analytical part specific to the variables we were interested in was not correct in three studies as described in our results section. The clinical trial was not rigorously analyzed using multivariate logistic regression.

### 2.5. Operational Definition

In this review, postpartum infection defining terminologies were perineal infection, maternal sepsis, surgical or traumatic wound infection, endometritis, urinary tract infection, too painful perineum, postpartum fever, septic pelvic thrombophlebitis, and peritonitis and mastitis after delivery within 6-week period. OVD reported as obstetric forceps or vacuum assisted vaginal delivery or trial was included. In this review, postpartum infection and maternal infection are interchangeably used.

### 2.6. Data Analysis and Presentation

The summary of the included studies, pertinent to the study's objective, is presented, highlighting the major findings and identifying risk factors for postpartum infection. Some data reported in tabular form for a different dependent variable are converted to bar graphs to show the absolute and relative risks of OVD for postpartum infection with other modes of delivery, including accompanied episiotomy and/or 3^rd^/4^th^ degree perineal tears.

Some additional bivariate analyses were done for the randomized clinical trial study taking the presented data to show the relative risk of postpartum infection and wound dehiscence comparing perineal wound infection and dehiscence with other types of maternal infection. One more bivariate analysis was done using the Acosta et al. study data [16] to show the statistically significant association of episiotomy with perineal infection.

Subgroup meta-analysis was done using OpenMetaanalyst software for Windows 10 (64-bit) to assess the odds ratios (OR) of OVD for postpartum infection in comparison with spontaneous vaginal delivery (SVD) and CS. The contribution of OVD among women who developed postpartum infection was also determined in the meta-analysis. Since there is significant heterogeneity among included studies, the random effect model was used. Included studies' heterogeneity was assessed by computing values for *I*^2^ and *P* values. The OR and 95% confidence intervals were computed with the DerSimonian–Laird method. Three-digit fraction numbers were approximated to two-digit numbers.

## 3. Results

**Figure 1:**
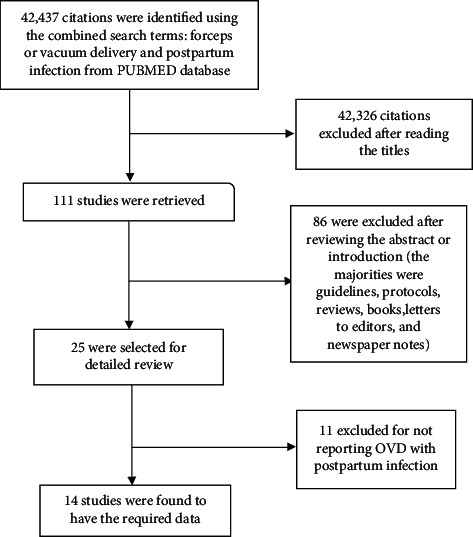
Flow diagram showing the selection process of the included studies (PRISMA).

As presented in Figure 1 (PRISMA flow chart), 14 articles were eligible for this review [2, 7, 14, 16–26]. One clinical trial and 13 observational studies (2 case-control and 11 cohort) were included (Table 1). All were from Western developed countries, predominantly from the United States of America and United Kingdom. The study populations in the majority of these studies (in the interest of this review) were in thousands, ranging from 400 to 65,991.

The risk of postpartum infection ranges from 0.1% to 18%. CS, episiotomy, and perineal tear were identified as a major risk factor for postpartum infection in the majority of the included studies. Nine studies performed logistic regression and determined the adjusted odds ratio; however, as described below, the major confounders (at least episiotomy and perineal tear) were not included in the adjusted analysis. The instrumental interventions were reported as forceps/vacuum trial [21, 22], applied [24], or delivery [19, 20].

Table 2 summarizes the association of maternal infection with OVD in the antibiotic prophylaxis and placebo groups [14]. This study was a multicenter randomized controlled trial with large sample size (1715 in the amoxicillin and clavulanic acid group and 1705 in the placebo group) aiming to investigate the efficacy of antibiotic prophylaxis in preventing maternal infection after OVD. In the original study, for the primary outcome variable (antibiotic prescription for confirmed or suspected maternal infection within 6 weeks of delivery, including perineal wound infection, endomyometritis, urinary tract infection, pyelonephritis, bacteremia, and sepsis), unadjusted risk ratios with 95% CI were determined.

It was reported that in 89% of the included study participants, episiotomies were performed. There were 1053 (31%) perineal tears (266 isolated and 787 with episiotomy). Overall, 97% (3310/3420) of the study participants had perineal wound for whom repairs were performed for almost all. Among women for whom episiotomy was done and/or who experienced perineal tear, 13.6% (414/3044) developed wound breakdown. Perineal wound sutured was the base for determining the isolated perineal tear (1645 − 1519 = 126; 1665 − 1525 = 140). Thus, the difference between the total and isolated perineal tears (493 − 126 = 367; 560 − 140 = 420) was the double perineal wound (episiotomy + perineal tear).

The risk of wound breakdown in the intervention and placebo group was 142 (11%) and 272 (21%), respectively. Interestingly, the proportion of this result was almost comparable with the risk of maternal infection in the intervention and placebo group, 180 (11%) and 306 (18%), respectively. With regard to the absolute risk, assuming that all wound breakdowns were due to infection, the remaining 38 (2.9%) maternal infections in the intervention group and 34 (2.6%) maternal infections in the placebo group were some other infections as listed as primary outcome indicators and were comparable. The absolute risk of ever too painful perineum in the antibiotic group was also comparable with an absolute risk of wound breakdown and overall maternal infection (11% each).

As a result, three variables (maternal infection other than perineal wound infection, maternal infection other than wound breakdown, and endometritis) did not show statistically significant difference between the antibiotic and placebo groups. The perineal wound infections accounted for 62% (111/180) of antibiotic group and 73% (222/306) of the placebo group. The implication is that the statistically significant difference between the antibiotic and placebo groups was because of the perineal wound infection. The absolute risk of perineal infection was incomparably higher than other types of maternal infections, including endometritis.

**Figure 2:**
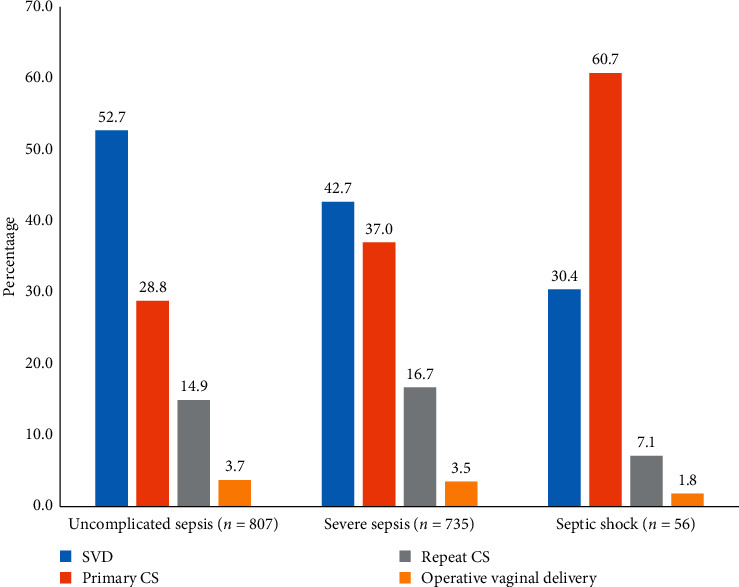
The relative risk of operative vaginal delivery to maternal sepsis as compared to other modes of delivery, redeveloped from Acosta et al.'s study 3 (*P* values for uncomplicated sepsis, severe sepsis, and septic shock in relation to mode of delivery were <0.0001, 0.001, and 0.006, respectively) (*N* = 1,622,474).

As shown in Figure 2, the relative risk of maternal sepsis and perineal infections was much lower in OVD as compared with other modes of delivery [19]. The relative risk of uncomplicated sepsis in OVD was about 14-fold, 8-fold, and 4-fold lower than SVD and primary and repeat CS, respectively. Similarly, the relative risks of severe sepsis and septic shock in OVD were much lower than other modes of delivery, which were highly statistically significant.

**Figure 3:**
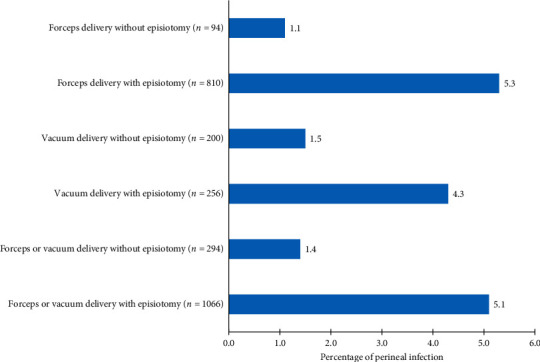
The relative risk of operative vaginal delivery to perineal infection as compared to episiotomy, redeveloped from Macleod et al.'s study (adjusted OR and 95% CI for vacuum and forceps with episiotomy were reported as 2.9 (0.81–10.71) and 5.2 (0.71–38.31); for all forceps and vacuum deliveries with episiotomy, *P*=0.002).

The absolute risk of perineal infection in forceps or vacuum delivery with episiotomy was more than 3.6-fold higher (5.1% vs 1.4%) than OVD conducted without episiotomy (Figure 3). The relative risk of perineal infection with episiotomy was also highly significant (odds ratios (OR) for vacuum and forceps were 2.9 and 5.2, respectively; *P* value of the overall = 0.002) [16].

**Figure 4:**
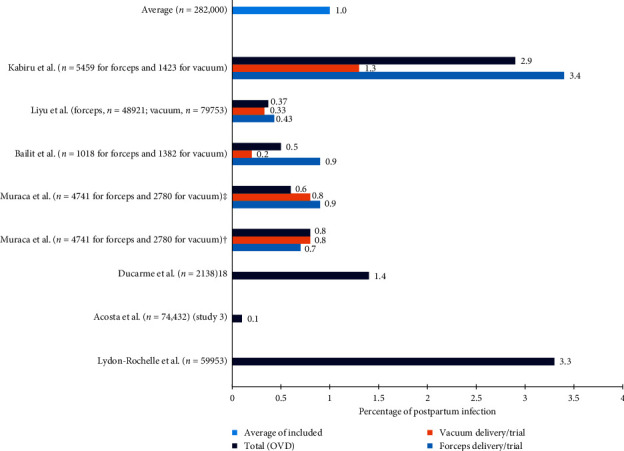
The proportion of postpartum infection (absolute risk) in large sample size studies among women for whom operative vaginal deliveries (OVD) were provided [17, 19, 20, 22]. ^†^Women with dystocia and prolonged second stage; ^‡^women with fetal distress and prolonged second stage.

As the different large sample size study reports are summarized in Figure 4, the absolute risk of developing postpartum infection was even much lower than the above reports (the majority of total OVD related postpartum infection ranging from 0.1% to 0.5%, and the highest was 3.3%), with an average of 1% of the included studies in the graph [19–22, 24–26]. In Ducarme et al.'s study [23], the risk of postpartum endometritis was compared between OVD and repeat CS without OVD attempt. The risk of endometritis at OVD was by more than 3.6-fold less common than CS.

In Gommesen et al.'s study [7], the overall risks of maternal infection and perineal wound dehiscence were 5.8% and 15.3%, respectively. The odds of maternal infection and perineal wound dehiscence in the episiotomy group were almost 3-fold and 1.6-fold higher than their counterparts. The risk of postpartum infection in 3^rd^/4^th^ degree perineal tear was three-times less than 2^nd^ degree tear; the common use of antibiotic prophylaxis in 3^rd^/4^th^ degree tears was attributed to the observed reduction. Perineal wound dehiscence was also by 68% less common among women who were given any antibiotic.

Axelsson et al.'s study reported that out of 795,072 deliveries at term, 11.7% women had infection in the postpartum period. Specifically, the risks of wound infection, endometritis, and urinary tract infection were 0.5%, 1.5%, and 0.2%, respectively. OVD was done for 65,991 (8.3%) deliveries. The distribution of wound infection, endometritis, and urinary tract infection by OVD was 0.8%, 1.8%, and 0.5%, respectively. The risk of endometritis was lower by 1.6-fold than CS. For seventeen variables (including OVD and CS), the statistical test was done against the gestational age of 37–40 weeks, for which almost all showed highly statistically significant association in both crude and adjusted analysis [2]. We are not clear why term pregnancy was taken as a reference for all the variables as a risk factor for infection.

Another study that has shown the extremely low incidence of major puerperal infection among readmitted postpartum women was reported by Liyu et al. [25]. The incidence rates among women who delivered by SVD, vacuum, forceps, and CS were 0.27%, 0.33%, 0.43%, and 0.45%. The relative risk (SVD as reference), however, has shown that puerperal infection was weakly associated with vacuum (OR = 1.2) and moderately associated with forceps (OR = 1.6) and CS (OR = 1.8). In Berghella and Bellussi's study [26], the incidence rates of uterine infection in the SVD, OVD, and CS were 2.9%, 3.3%, and 5.2%, respectively, which were again weakly significant for OVD (OR = 1.2) and strongly significant for CS (OR = 5.2).

Two case-control studies were included to show the contribution of OVD to the selected cases among the different modes of delivery. Acosta et al.'s study 1 has shown that out of 89 uncomplicated sepsis cases, 32.6%, 19.1%, and 48.3% accounted for SVD, OVD, and CS, respectively. In the control group, the distribution of SVD, OVD, and CS was 14.3%, 21.4%, and 64.3%, respectively. The unadjusted OR and adjusted OR with 95% CIs for OVD were reported as 1.13 (0.59–2.15) and 2.20 (1.02–4.87), respectively [17]. But we could not understand how the AOR reported as statistically significant with the given data (taking SVD as a reference) and OVD was interpreted as a risk factor for uncomplicated maternal sepsis. The proportion of severe sepsis among OVDs was 1.5-fold higher and 3-fold less than SVD and CS cases, respectively, but was not statistically significant.

Similarly, Acosta et al.'s study 2 reported that the proportion of OVD in cases/severe sepsis and controls was 14.5% and 13.3%, respectively. In the same category, the case/control distribution of SVD, elective CS, and emergency CS was 25.5%/58.8%, 22%/15.8%, and 38%/12.2%, respectively. The UOR and AOR with 95% CIs for OVD were reported as 2.52 (1.52–4.17) and 2.49 (1.32–4.70), respectively [18]. We posed here as well the same question: how the statistical test showed statistical significance for OVD as a risk factor while the proportion was lower than SVD and CS, and with little difference between cases and controls? Interestingly, in both study 1 and 2 [17, 18], the proportion of the overall maternal sepsis among women delivered assisted by instruments was much lower than SVD and CS (19.4% and 14.5%).

**Figure 5:**
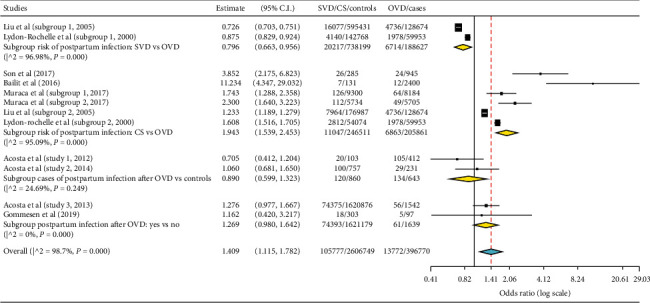
Subgroup meta-analysis of postpartum infection risk after operative vaginal delivery (OVD) in comparison with spontaneous vaginal delivery (SVD) and caesarean section (CS) delivery. The subgroup meta-analysis was done for SVD vs OVD, CS vs OVD, and the contribution of OVD to cases or postpartum infection.

The subgroup meta-analysis could not also show statistically significant association in both these two studies (Figure 5). Similarly, the risk of postpartum infection among women who delivered by OVD as compared with other modes of delivery (the last row of subgroup meta-analysis) was not significant.

Weak association of OVD with postpartum infection was observed in the SVD vs OVD subgroup meta-analysis (OR = 0.8; 95% CI, 0.66–0.96). The odds of postpartum infection in women who delivered by CS were close to 2-fold higher than OVD (OR = 1.9; 95% CI, 1.52–2.45). The pooled meta-analysis demonstrated that the majority of postpartum infection occurred in the non-OVD groups (OR = 1.4; 95% CI, 1.12–1.78). With the exclusion of CS vs OVD studies (as sensitivity analysis), there was no statistical significant difference in the pooled meta-analysis (OR = 0.89; 95% CI, 0.75–1.05).

## 4. Discussion

This review and meta-analysis could not find a substantial evidence from either the randomized controlled trial or observational studies to routinely use prophylactic antibiotics at OVD. The analysis clearly demonstrated the increased risk of postpartum infection after OVD when the procedure was accompanied by surgical or traumatic injury to the perineal area.

Knight and colleagues recommended to WHO, the UK, North America, and Australia to change their guidelines (which neither recommends antibiotic prophylaxis at OVD) by stating that a single dose of prophylactic antibiotic at OVD has reduced the risk of maternal infection by about half [14]. Berghella and Bellussi's comment on this study was quite positive and persuasive; they expressed their view as the study finding is a “practice-changing data” [27]. American College of Obstetricians and Gynecologists (ACOG) abstract has also concluded positively by stating that this trial provides robust evidence for the use of routine prophylactic antibiotics at OVD [28].

However, as the subgroup analysis of that particular study showed, cases which had shown statistically significant difference in the antibiotic group were perineal wound infections, wound breakdown, and ever too painful perineum, for which the episiotomy and perineal tear wound were the main risk factors, not the OVD per se. Had the investigators done multivariate analysis of maternal infection in the intervention and placebo group taking OVD, episiotomy, and perineal tear as independent variables, only the latter two would have shown a statistically significant association.

The finding of comparative result between the two groups in nonperineal wound infection, endometritis, and maternal infection other than wound breakdown reaffirms the significance of surgical and traumatic perineal wound for the increased risk of perineal wound infection and the importance of prophylactic antibiotic at OVD accompanied by perineal trauma. Although perineal wound infection and wound breakdown were reported with different absolute figure, our argument is that, until proved otherwise, almost all wound breakdown cases were due to episiotomy and perineal tear wound infection, predisposed by the surgical and traumatic wound, not merely by the applied instruments.

In line with this, a Cochrane review defined episiotomy wound dehiscence as wound infection [29]. A recent prospective cohort study from Denmark has also shown the complications of OVD (increased risk of perineal tears) and episiotomy (3-fold increased risk of infection/wound dehiscence) [7]. The point is nearly all the study participants included in this trial had one or more much higher risk factors for postpartum infection than instrument application, which were not corrected by randomization or multivariate analysis.

In our opinion, this is enough evidence to deduce that the study [14] was actually a comparison of antibiotic prophylaxis with placebo in women with episiotomy and perineal tear wound, as 97% of the study participants had either one or both and as the bivariate statistical tests clearly demonstrated the significant association with only perineal wound infection, perineal wound breakdown, and ever too painful perineum.

Whether to give antibiotic prophylaxis or not in women with episiotomy is one of the controversial issues in obstetrics; WHO, ACOG, and others are against it [30–33]; a Cochrane review weakly recommended it [34]. This study [14], however, has demonstrated the statistically significant difference in wound breakdown and perineal wound infection between the intervention and the placebo group, which is an important finding to consider prophylactic antibiotic after episiotomy and perineal tear.

Nevertheless, it would have been good if the statistical analysis was not limited only to bivariate analysis; although it is a randomized trial which gave equal chance to the potential confounders other than the perineal wound, multivariate analysis could have been better to further control the confounders and increase the strength of evidence, particularly in light of multiple risk factors for perineal wound infection.

With the exception of Gommesen et al.'s study [7], other studies included in this review as well assessed the risk of postpartum infection mainly by comparing OVD with other modes of delivery without adjusting to the surgical and traumatic perineal wound; some authors even tempted to use prophylactic antibiotics at OVD [17]. The condition was that many of the OVDs used to require episiotomy and got complicated with perineal tear. Such interconnectedness of interventions and associated traumatic complication was probably the main reason for wrongly attributing postpartum infection to OVD. A literature review by Kamel and Khaled overemphasized the association of postpartum infection with OVD without giving emphasis to the commonly associated episiotomy and perineal tear [35].

Acosta et al.'s study [16], however, has shown about 3-fold and 5-fold independent association of perineal infection in women who delivered assisted by vacuum or forceps after episiotomy, respectively. In their study, the incidence of perineal infection after forceps and vacuum delivery without episiotomy was extremely low. Those who developed perineal infection without episiotomy were those who had perineal tear, as perineal infection in intact perineum is very unlikely. Although the sample size was relatively small, Gommesen et al.'s study also clearly showed the absence of statistically significant association of OVD with postpartum infection. The meta-analysis finding in this study is consolidating evidence to the above arguments.

Above all, the extremely low absolute risk of postpartum infection (on average 1%) at OVD in many of the large sample size studies which had reported the incidence of postpartum infection over three decades and the very weak relative risk in a few studies [2, 25] are not robust evidence to consider prophylactic antibiotic at OVD without perineal wound. This is because antibiotic prophylaxis at OVD and antibiotic prophylaxis at episiotomy and perineal tear are different clinical settings. The essence is that as all OVDs do not require episiotomy and do not complicate with a perineal tear as shown in some of the included studies [16, 20, 21], there is no supporting evidence to routinely administer prophylactic antibiotic for mere application of instruments to assist vaginal delivery.

In the included clinical trial [14], the highly increased absolute risks of maternal infection and wound breakdown in both antibiotic and placebo groups (more than 10-fold of the incidence of cohort studies) were probably due to the fact that almost every study participant had either episiotomy, perineal tear, or both. In other words, instrument assisted delivery is known to increase the risk of perineal tear and genital laceration, but not directly and necessarily mean that it increases wound infection.

Episiotomy and perineal tear wound infections can occur without instrument-assisted vaginal delivery [36]; although reported data showed contradictory results, increased risk of perineal wound infection is because of iatrogenic or natural birth injury to the genital tract, which is susceptible to contamination by the normal flora of the birth canal and anal canal [37]. A prospective multicenter study has also showed that the source of bacteria for antepartum, intrapartum, and postpartum maternal infection was the genital tract in 61% of patients, predominantly *Escherichia coli*, followed by Group B Streptococcus [38].

Therefore, the available scientific data this time around do not have the leverage to change the existing guidelines and practice at OVD without episiotomy and without a perineal tear. Routine use of prophylactic antibiotic at OVD may unnecessarily increase the risk of antibiotic resistance, as the high incidence of infection noted in the clinical trial and progressively increasing nonsusceptibility of the commonest bacterial etiology (Escherichia coli) of postpartum infection to amoxicillin and clavulanic acid were reported [39].

The increased risks of perineal wound infection and perineal wound breakdown in both the randomized clinical trial and cohort studies are, however, indicative data to consider prophylactic antibiotic for episiotomy and perineal tear until another clinical trial proved it otherwise.

We recommend that the clinical trial with antibiotic prophylaxis at OVD has to be done in women without iatrogenic or natural genital trauma with a hypothesis of introducing bacteria while applying instruments can increase the risk of postpartum maternal infection. Otherwise, in the presence of an already established risk factor for maternal infection (major epithelial and muscle breakdown requiring repair in the perineal area), attributing OVD to it sounds little and will not convince clinicians to change their current practice.

One of the limitations of this review was lack of clinical trial investigating the risk of OVD to postpartum infection without perineal trauma. The second is the majority of the included studies did not include episiotomy and perineal tear as independent variables for multivariate analysis.

In conclusion, both the relative and absolute risks of postpartum infection at OVD are extremely lower than the caesarean delivery and delivery accompanied by episiotomy and 3^rd^/4^th^ degree perineal tear. Thus, from previous studies, there is no subtantial evidence to use prophylatic antibiotic at OVD, but women with episiotomy and perineal tear can benefit from prophylactic antibiotic administration.

## Figures and Tables

**Table 1:** General characteristics of the included studies in relation to postpartum infection.

Authors/study period/country	Study design	Study population	Risk of postpartum infection	Risk factors attributed	Statistical analysis done for
Knight et al./March 13, 2016—June 13, 2018, UK	Multicenter randomized clinical trial	1715 trial and 1705 placebo group	11% trial group and 18% placebo group	Operative vaginal deliveries (OVD) (forceps and vacuum)	Unadjusted risk ratio with 95% CI

Acosta et al./1986–2009 (study 1), Scotland	Case-control	103 cases and 412 controls	Not reported	Obesity, age <25 years, OVD, multiparity, anemia, labor induction, CS, and preterm	AOR with 95% CI

Acosta et al./June 1, 2011—May 31, 2012 (study 2), UK	Case-control	365 cases and 757 controls	Incidence of severe sepsis, 4.7 per 10,000 maternities	Black or other ethnic minority, primiparous, preexisting illness, or on antibiotics, OVD, and CS	AOR with 95% CI

Acosta et al./2005–2007 (study 3), USA	Retrospective cohort	1598 women with sepsis	Incidence of all sepsis, 10 per 10,000 live births	PPH, hypertension, multiple birth, primiparous, black	AOR with 95% CI

Macleod et al./Oct 2004–Aug 2006/UK	Prospective cohort	1366	Perineal infection in episiotomy group 5.1%	Vacuum and forceps delivery with episiotomy	AOR with 95% CI

Kabiru et al./1980–1996/USA	Retrospective cohort	6882	2.9%	Not reported	Unadjusted relative risk with 95% CI

Bailit et al./2008–2011/USA	Retrospective cohort	2531	0.5% with OVD and 5.3% with CS	Strongly CS, weakly OVD	AOR with 95% CI

Muraca et al./2003–2013/Canada^†^	Retrospective cohort	8184 OVD, 9300 CS	0.8% with OVD and 1.4% with CS	CS	AOR with 95% CI

Muraca et al./2003–2013/Canada^‡^	Retrospective cohort	5705 OVD, 5734 CS	0.9% with OVD and 2.0% with CS	CS	AOR with 95% CI

Son et al./4 years/USA	Retrospective analysis	945 OVD, 285 repeat CS	Endometritis: 2.5% with OVD and 9.1% with CS	CS	AOR with 95% CI

Ducarme et al./Dec 2008–Oct2013/France	Prospective cohort	2138	1.4%	Not tested specific to maternal infection	NA

Gommesen et al./Jul 2015–Jan 2018/Denmark	Prospective cohort	400	Infection: 5.8%; perineal wound dehiscence:15.3%	Episiotomy and obesity	AOR with 95% CI

Axelsson et al./2005–2012/Sweden	Retrospective cohort	5,991	Overall infection: 11.7%	CS, perineal tear, episiotomy, anemia, placenta removal, OVD	AOR with 95% CI

Lydon-Rochelle et al./1987–1996/USA	Retrospective cohort	256,795	Overall uterine infection rate: 3.5%	Strongly CS, weakly OVD	AOR with 95% CI

Liyu et al./1997–2001/Canada	Retrospective cohort	900,108	Major puerperal infection: 0.3%	Weakly with vacuum and a bit strongly with forceps and CS	AOR with 95% CI

^†^Women with dystocia and prolonged second stage; ^‡^women with fetal distress and prolonged second stage.

**Table 2:** Obstetric procedures and traumatic and infectious complications (redeveloped from the original study by Knight et al. with some additional variables and analysis).

Procedures and complications	Antibiotic trial group (*n* = 1715), no (%)	Placebo group (*n* = 1705), no (%)	*P* value
Episiotomy (total)	1519 (89.0)	1525 (89.0)	0.2
Perineal tear (total)	493 (29.0)	560 (33.0)	0.004
Isolated perineal tear	126 (7.0)	140 (8.0)	0.2
Perineal tear and episiotomy	367 (21.0)	420 (25.0)	0.01
Sutured perineal wound	1645 (99.0)^*∗*^	1665 (100.0)^*∗*^	NA
Confirmed or suspected maternal infection	180 (11.0)	306 (18.0)^‡^	<0.0001
Perineal wound infection	111 (7.0)	222 (13.0)	<0.0001
Maternal infection other than perineal wound infection	69 (4.0)	84 (5.0%)	0.1
Wound breakdown^†^	142 (11.0)	272 (21.0)	<0.0001
Maternal infection other than wound breakdown	38 (2.0)	34 (2.0)	0.2
Endometritis	15 (1.0)	23 (1.0)	0.2
Ever too painful perineum^†^	136 (11.0)	198 (15.0)^‡^	<0.00025

^†^The denominator for antibiotic trial and placebo groups is 1296 and 1297, respectively. ^‡^The percentage in the original study for two variables in the placebo group (“confirmed or suspected maternal infection” and “ever too painful perineum”) was reported as 19% and 17%, respectively. We thought that as a typographic error and made corrections. ^*∗*^The “missing” cases (54 for antibiotic trial group and 33 for the placebo group) were included in numerator in the original study report to determine the proportion, giving 99% and 100%, respectively.

## Supplementary Materials

Supplementary Materials PRISMA checklist and STROBE check list.
