# Supplementary material for: Still No Substantial Evidence to Use Prophylactic Antibiotic at Operative Vaginal Delivery: Systematic Review and Meta-Analysis
Source: Obstet Gynecol Int. 2020 May 19;2020:1582653. doi: 10.1155/2020/1582653 (PMC7479451; doi:10.1155/2020/1582653)
Supplement: Supplementary Materials — PRISMA checklist and STROBE check list. [file 1582653.f1.pdf]

PRISMA 2009 Checklist (Adapted for KIN 4400)

Section/topic	#	Checklist item	Reported on page #
TITLE: **Still no substantial evidence to use prophylactic antibiotic at operative vaginal delivery: a systematic review**			
Title	1	Identify the report as a literature review.	1
**ABSTRACT**			
Structured summary	2	Provide a structured summary including, as applicable: background; objectives; data sources; study eligibility criteria, participants, and interventions; study appraisal and synthesis methods; results; limitations; conclusions and implications of key findings;	2
**INTRODUCTION**			
Rationale	3	Describe the rationale for the review in the context of what is already known about your topic.	3-4
Objectives	4	Provide an explicit statement of questions being addressed with reference to participants, interventions, comparisons, outcomes, and study design (PICOS).	4
**METHODS**			
Eligibility criteria	5	Specify study characteristics (e.g., PICOS, length of follow-up) and report characteristics (e.g., years considered, language, publication status) used as criteria for eligibility, giving rationale.	5
Information sources	6	Describe all information sources (e.g., databases with dates of coverage) in the search and date last searched.	5
Search	7	Present full electronic search strategy for at least one database, including any limits used, such that it could be repeated.	5
Study selection	8	State the process for selecting studies (i.e., screening, eligibility).	5-6
Risk of bias in individual studies	9	Describe methods used for assessing risk of bias of individual studies (including specification of whether this was done at the study or outcome level).	6
Risk of bias across studies	10	Specify any assessment of risk of bias that may affect the cumulative evidence (e.g., publication bias, selective reporting within studies).	6
**RESULTS**			
Study selection	11	Give numbers of studies screened, assessed for eligibility, and included in the review, with reasons for exclusions at each stage, ideally with a flow diagram.	8
Study characteristics	12	For each study, present characteristics for which data were extracted (e.g., study size, PICOS, follow-up period) and provide the citations.	8

PRISMA 2009 Checklist (Adapted for KIN 4400)

Section/topic	#	Checklist item	Reported on page #
Synthesis of results of individual studies	13	For all outcomes considered (benefits or harms), present, for each study: (a) summary of results and (b) relationship to other studies under review (e.g. agreements or disagreements in methods, sampling, data collection or findings).	8-12
**DISCUSSION**			
Summary of evidence	14	Summarize the main findings including the strength of evidence for each main outcome; consider their relevance to key groups (e.g., healthcare providers, users, and policy makers).	12-16
Limitations	15	Discuss limitations at study and outcome level (e.g., risk of bias), and at review-level (e.g., incomplete retrieval of identified research, reporting bias).	17
**CONCLUSION**			
Conclusions	16	Provide a general interpretation of the results in the context of other evidence, and implications for future research.	17

Adapted from: Moher D, Liberati A, Tetzlaff J, Altman DG, The PRISMA Group (2009). Preferred Reporting Items for Systematic Reviews and Meta-Analyses: The PRISMA statement. *PLoS Medicine*, 6(6), e1000097. doi:10.1371/journal.pmed1000097

For more information, visit: **www.prisma-statement.org**.

Title of the article: **Still no substantial evidence to use prophylactic antibiotic at operative vaginal delivery: systematic review and meta-analysis**

STROBE guidelines

Section/topic	Item number	Recommendation
Title and abstract	1	Indicates the study's design with a commonly used term in the title or the abstract<br>Provide in the abstract an informative and balanced summary of what was done and what was found
Introduction		
Background/rationale	2	Explains the scientific background and rationale for the investigation being reported
Objectives	3	States specific objectives, including any prespecified hypotheses
Methods		
Study design	4	Presents key elements of study design early in the manuscript
Setting	5	Describes the setting, locations, and relevant dates, including periods of recruitment, exposure, follow-up, and data collection
Participants	6	Cohort study - gives the eligibility criteria, and the sources and methods of selection of participants; describe methods of follow-up<br>Case-control study - gives the eligibility criteria, and the sources and methods of case ascertainment and control selection; gives the rationale for the choice of cases and controls<br>Cross-sectional study - gives the eligibility criteria, and the sources and methods of selection of participants<br>Cohort study - for matched studies, gives matching criteria and number of exposed and unexposed Case-control study - for matched studies, gives matching criteria and the number of controls per case
Variables	7	Clearly defines all outcomes, exposures, predictors, potential confounders, and effect modifiers; give diagnostic criteria, if applicable
Data sources/measurement	8	For each variable of interest, gives sources of data and details of methods of assessment (measurement); Describes comparability of assessment methods if there is more than one group
Bias	9	Describes any efforts to address potential sources of bias
Study size	10	Explains how the study size was arrived at
Quantitative variables	11	Explains how quantitative variables were handled in the analyses; if applicable, describes which groupings were chosen and why
Statistical methods	12	Describes all statistical methods, including those used to control for confounding

Section/topic	Item number	Recommendation
		Describe any methods used to examine subgroups and interactions<br>Explains how missing data were addressed<br>Cohort study - if applicable, explains how loss to follow-up was addressed<br>Case-control study - if applicable, explains how matching of cases and controls was addressed<br>Cross-sectional study - if applicable, describes analytical methods taking account of sampling strategy<br>Describe any sensitivity analyses
Results		
Participants	13	Reports numbers of individuals at each stage of study - e.g., numbers potentially eligible, examined for eligibility, confirmed eligible, included in the study, completing follow-up, and analyzed<br>Give reasons for nonparticipation at each stage Consider use of a flow diagram
Descriptive data	14	Gives characteristics of study participants (e.g., demographic, clinical, social) and information on exposures and potential confounders<br>Indicate number of participants with missing data for each variable of interest Cohort study - summarize follow-up time (e.g., average and total amount)
Outcome data	15	Cohort study - reports numbers of outcome events or summary measures over time<br>Case-control study - report numbers in each exposure category, or summary measures of exposure<br>Cross-sectional study - reports numbers of outcome events or summary measures
Main results	16	Gives unadjusted estimates and, if applicable, confounder-adjusted estimates and their precision (e.g., 95% confidence interval); make clear which confounders were adjusted for and why they were included Report category boundaries when continuous variables were categorized<br>If relevant, consider translating estimates of relative risk into absolute risk for a meaningful time period
Other analyses	17	Reports other analyses done - e.g., analyses of subgroups and interactions, and sensitivity analyses
Discussion		
Key results	18	Summarizes key results with reference to study objectives
Limitations	19	Discusses limitations of the study, taking into account sources of potential bias or imprecision; discuss both direction and magnitude of any potential bias
Interpretation	20	Gives a cautious overall interpretation of results considering objectives, limitations, multiplicity of analyses, results from similar studies, and other relevant evidence
Generalizability	21	Discusses the generalizability (external validity) of the study results

Section/topic	Item number	Recommendation
Other information		
Funding	22	Gives the source of funding and the role of the funders for the present study and, if applicable, for the original study on which the present article is based

Table. Risk of bias assessment using STROBS (Strengthening the Reporting of Observational Studies) checklist.

Section/topic	Item number	Acosta et al (Study 1)	Acosta et al (Study 2)	Acosta et al (Study 3)	Macleod et al	Kabiru et al	Bailit et al	Muraca et al	Son et al	Ducarme et al	Gommesen et al	Axelsson et al/	Lydon-Rochelle et al	Liyu et al
Title and abstract	1	√	√	√	√	√	√	√	√	√	√	√	√	√
Introduction														
Background /rationale	2	√	√	√	√	√	√	√	√	√	√	√	√	√
Objectives	3	√	√	√	√	√	√	√	√	√	√	√	√	√
Methods														
Study design	4	√	√	√	√	√	√	√	√	√	√	√	√	√
Setting	5	√	√	√	√	√	√	√	√	√	√	√	√	√
Participants	6	√	√	√	√	√	√	√	√	√	√	√	√	√
Variables	7	√	√	√	√	√	√	√	√	√	√	√	√	√
Data sources /measurement	8	√	√	√	√	√	√	√	√	√	√	√	√	√
Bias	9	√*	√*	x	√*	√*	√	√*	x	x	√*	√*	x	x
Study size	10	√	√	√	√	√	√	√	√	√	√	√	√	√
Quantitative variables	11	√	√	√	√	√	√	√	√	√	√	√	√	√
Statistical methods	12	√	√	√	√	√	√	√	√	√	√	√	√	√
Results														

Participants	13	√	√	√	√	√	√	√	√	√	√	√	√	√
Descriptive data	14	√	√	√	√	√	√	√	√	√	√	√	√	√
Outcome data	15	x	x	√	√	√	√	√	√	√	√	x	√	√
Main results	16	x	x	√	√	√	√	√	√	√	√	x	√	√
Other analyses	17	√	√	√	√	√	√	√	√	√	√	√	√	√
Discussion														
Key results	18	√	√	√	√	√	√	√	√	√	√	√	√	√
Limitations	19	√	√	√	√	√	√	√	√	√	√	√	√	√
Interpretation	20	√	√	√	√	√	√	√	√	√	√	√	√	√
Generalizability	21	√	√	√	√	√	√	√	√	√	√	√	√	√
Other information														
Funding	22	√	√	√	√	√	√	√	√	√	√	√	√	√

* Potential sources of bias mentioned in the discussion section or as a limitation of the study
